# Project ART-ED: alcohol reduction and HIV testing in the emergency department

**DOI:** 10.1186/1940-0640-7-S1-A35

**Published:** 2012-10-09

**Authors:** E Jennifer Edelman, An Dinh, Lucian Radu-Radulescu, Bonnie Lurie, Jeanette Tetrault, Gail D'Onofrio, David Fiellin, Lynn Fiellin

**Affiliations:** 1 Veterans Affairs Connecticut Healthcare System Clinical Scholar's Program and Yale University, New Haven, CT, USA; 2 Yale University School of Medicine, New Haven, CT, USA; 3 Substance Abuse Treatment Program, Veterans Affairs Central Western Massachusetts, Pittsfield, MA, USA

## 

Unhealthy alcohol use and HIV risk often co-occur. To intervene on this association, we are conducting a pilot study to determine the feasibility and impact of providing brief alcohol- and sexual-risk reduction counseling with rapid HIV testing in a large urban emergency department (ED). We are recruiting ED patients aged 18-40 years who 1) meet National Institute on Alcohol Abuse and Alcoholism criteria for at-risk drinking, 2) have >1 sexual-risk behavior, 3) have negative or unknown HIV status, and 4) are willing to undergo HIV testing. We are conducting a brief, manual-guided intervention combining an alcohol- and sexual-risk reduction counseling session with rapid HIV testing followed by a booster telephone call at two weeks. At baseline and eight weeks, we assess alcohol consumption with the Timeline Follow-Back for alcohol consumption and a modified HIV Risk Behavior Scale to characterize sexual risk behaviors. Statistical analyses include Wilcoxon Signed Rank test, McNemar test, and two-way ANOVA. Of the 82 participants enrolled to date, 60% are male, the mean age is 25 years, 63% are white, 83% are unmarried, 59% are college-educated, 41% are without primary care, and 79% have an AUDIT score of >8. All tested HIV negative. Among the 62 with follow-up data so far, alcohol consumption decreased with fewer average weekly drinks (25.5 versus 10.4, p < 0.0001) and binge drinking episodes (2.03 versus 0.99, p < 0.0001). This decrease was greater in men than women (p < 0.0002). Post-intervention, participants endorsed increased condom use (median change = 3 points on a 5-point scale, W = 275, p < 0.0001) and decreased episodes of sex while intoxicated (RR = 0.14, p < 0.0001). Mean intervention duration was 44 minutes. Preliminary analyses demonstrate that a brief intervention combining alcohol- and sexual-risk reduction counseling with rapid HIV testing in the ED is feasible and effective for reducing alcohol use and HIV risk behaviors among young unhealthy drinkers.

